# Rhabdomyolysis and Acute Renal Failure after Gardening

**DOI:** 10.1155/2015/174892

**Published:** 2015-04-12

**Authors:** Zeljko Vucicevic

**Affiliations:** Department of Internal Medicine, Intensive Care Unit, School of Medicine, “Sestre Milosrdnice” University Hospital Centre, University of Zagreb, Vinogradska Cesta 29, 10000 Zagreb, Croatia

## Abstract

Acute nontraumatic exertional rhabdomyolysis may arise when the energy supply to muscle is insufficient to meet demands, particularly in physically untrained individuals. We report on a psychiatric patient who developed large bruises and hemorrhagic blisters on both hands and arms, rhabdomyolysis of both forearm muscles with a moderate compartment syndrome, and consecutive acute renal failure following excessive work in the garden. Although specifically asked, the patient denied any hard physical work or gardening, and heteroanamnestic data were not available. The diagnosis of rhabdomyolysis was easy to establish, but until reliable anamnestic data were obtained, the etiology remained uncertain. Four days after arrival, the patient recalled working hard in the garden. The etiology of rhabdomyolysis was finally reached, and the importance of anamnestic data was once more confirmed.

## 1. Introduction

Acute nontraumatic exertional rhabdomyolysis may arise in individuals with normal muscles when the energy supply to muscles insufficiently meets demands [1]. Examples usually include ultramarathon races which may be aggravated by extremely hot, humid conditions particularly in physically untrained individuals [2]. Friction blisters on the palms and fingers frequently follow vigorous physical work or repetitive physical activities causing detachment of the skin epidermis. In more severe cases, particular areas of the skin can be entirely detached from the basis and blisters filled with blood. We report on a psychiatric patient who developed large bruises and hemorrhagic blisters on both hands and arms, rhabdomyolysis of both forearm muscles with a moderate compartment syndrome, and a consecutive acute renal failure following excessive work in the garden.

## 2. Case Report

**Figure 1:**
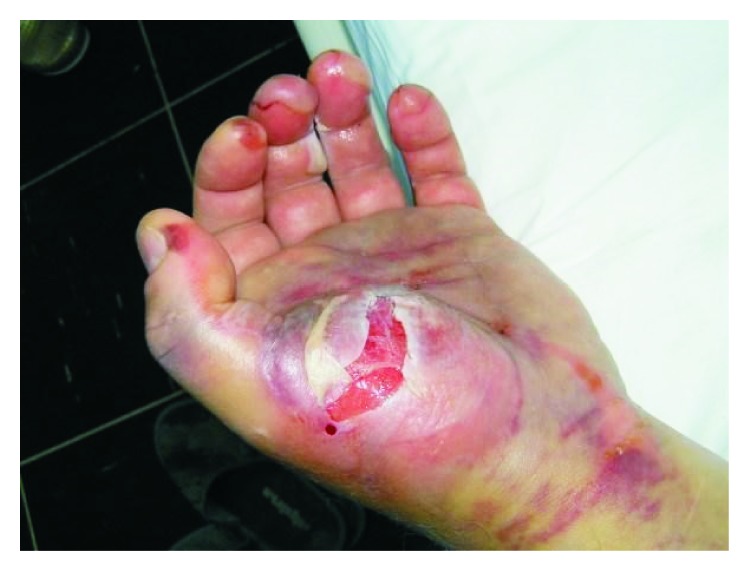
Hemorrhagic and nonhemorrhagic bullae, hematomas, and edema of the left palm.

**Figure 2:**
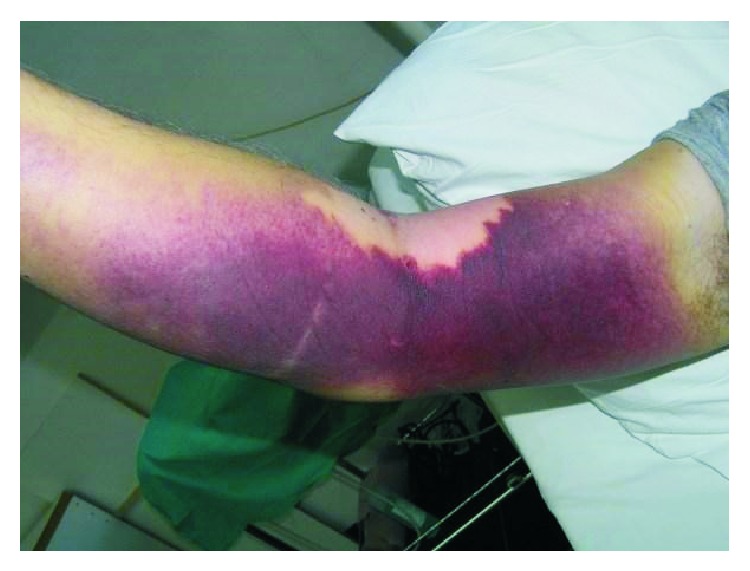
Large bruise and edema of the right arm and forearm.

A 55-year-old man was admitted to the intensive care unit because of bullae and hematomas of both hands and arms of unknown etiology (Figures 1 and 2).

Forearms were tense and swollen with large bruising of the skin. Hemorrhagic and nonhemorrhagic bullae were seen on the left palm and fingers. The patient was not capable of squeezing his left hand or of moving his fingers which was indicative of moderate compartment syndrome and nerve paresis. A few reddish skin indurations were visible on the front chest wall with no itching or burning sensation.

The patient had a history of long-term psychosis treatment with clozapine, haloperidol, alprazolam, and biperiden without other comorbidities. Upon arrival, he was alert, afebrile, eupneic, and normotensive and in a good general condition. No other abnormalities were noted at physical examination. The patient denied any trauma, hard physical work, contacts with chemicals, burns, cold, or alcohol and narcotic abuse. He also specifically denied any close contact with various kinds of plants or grasses.

Results of laboratory tests were as follows: C-reactive protein, red and white blood cell count, ECG, X-ray chest, blood sugar, electrolytes (K, Na, Cl, Ca, Mg, and P), and coagulation tests were within normal range. Results that lie outside the laboratory reference ranges are summarized in Table 1.

**Figure 3:**
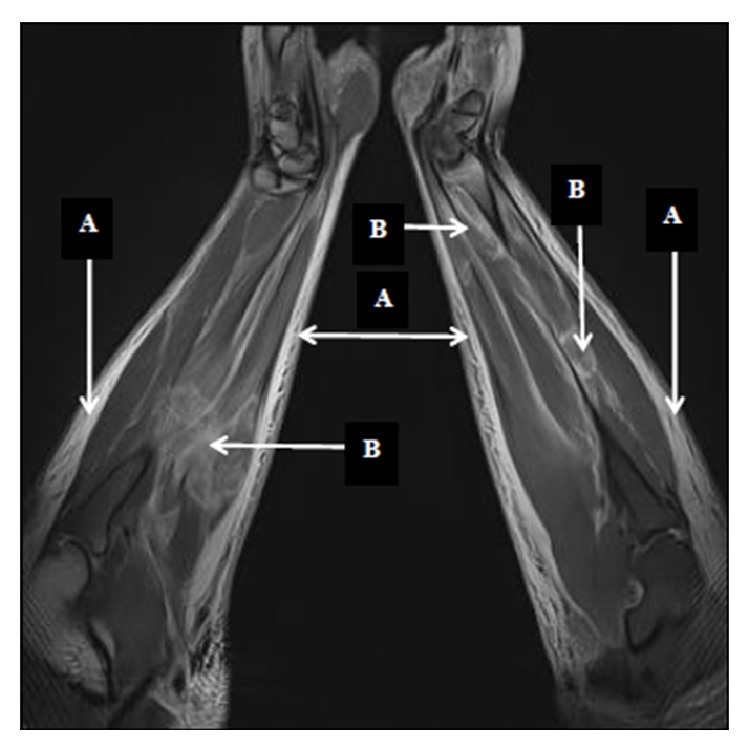
Coronal fat-suppressed proton density-weighted MR images of both forearms show diffuse hyperintensity in the subcutaneous fat tissue (A) and multifocal, confluent areas of hyperintensity in the muscles due to edema (B).

These laboratory findings were indicative of rhabdomyolysis and acute renal failure. The acid-base status of the capillary blood sample was still satisfying. Doppler ultrasound exam of both arms showed a normal arterial and venous blood flow. Nuclear magnetic resonance of both forearms revealed a diffuse edema of subcutaneous fat tissue and an extreme edema of forearm muscles (Figure 3).

The result of a chest and hand skin biopsy obtained later was unspecific and inconclusive. Bacteriological blister swabs were sterile. Serologic tests to antinuclear antibodies (ANA), rheumatoid factor (RF), and anti-Jo1 were negative.

The therapy consisted of an intensive fluid replacement, forced diuresis, antihistamine, and a surgical wound care.

On the fourth day of hospitalization, the patient suddenly recalled that he was pruning hedges with very small scissors and was pulling weeds with bare hands only 24 h before arrival to the hospital. On a hot day, stripped to the waist, he was entering into the bushes to reach the vegetation he wanted to cut, exposing his chest to pricking and prodding of sharp twigs and sprigs. After several hours of such a hard work, he noticed a few blisters and scratches on his hands, put the gloves on, and continued to work.

The five-day symptomatic treatment resulted in a moderate edema regression and completely restored renal function, and the patient was discharged. The full recovery occurred three weeks later.

## 3. Discussion

Bullae and blisters can be caused by various mechanical or environmental factors. One of the most known causes is friction that comes from using a various shovel or like in this case from grabbing hedges and weeds. The skin was directly traumatised and exposed to various flora, some of which might have had toxic effect. Such an extreme physical activity is known to result in some degree of rhabdomyolysis.

Numerous causes of rhabdomyolysis can be classified into three main categories: traumatic, nontraumatic exertional (e.g., marked exertion in untrained individuals), and nontraumatic nonexertional (e.g., drugs or toxins, infections, or electrolyte disorders) [3].

Nontraumatic exertional rhabdomyolysis may follow significant physical exertion in physically untrained persons especially in extremely hot and/or humid conditions [4]. We believe that was what happened with our patient.

Hypokalemia caused by potassium loss from sweating may additionally induce impairment of muscle metabolism and contribute to muscle dysfunction [5], but in our patient the serum potassium level was normal.

Once it happens, rhabdomyolysis may lead to kidney failure. There are several mechanisms by which rhabdomyolysis impairs the glomerular filtration rate. The pathogenesis of rhabdomyolysis is based on muscle cell death, depletion of adenosine triphosphate (ATP), and an increase in free ionized calcium in the cytoplasm [6]. ATP depletion causes dysfunction of the Na/K-ATPase and Ca^2+^ATPase pumps that are essential to maintaining integrity of the myocyte.

The lesion of skeletal muscle results in subsequent release of toxic intracellular contents into the circulatory system including myoglobin, creatine phosphokinase, potassium, aldolase, lactate dehydrogenase, and glutamic-oxaloacetic transaminase [7]. Experimental evidence suggests that intrarenal vasoconstriction, direct and ischemic tubule injury, and tubular obstruction all play a role [6].

One of the possible complications of rhabdomyolysis is the compartment syndrome. If the energy-dependent transcellular pump systems fail in the traumatized tissue, the muscle cells swell. As a result, intracompartmental pressure rises and may provoke additional myocyte damage and necrosis [8].

Although more common in the anterior compartment of the lower leg, it has been described in the forearm of motocross racers [9] and in an elite flatwater sprint kayaker [10].

For the first couple of days, our patient was unable to squeeze his left hand or move his fingers as a consequence of muscle edema and nerve compression typical of the compartment syndrome.

The key problems in this case were unreliable anamnestic data taken from the psychiatric patient who lived alone and heteroanamnestic data that were not available. Since the clinical features were limited to both arms, we primarily suspected a working contact with potentially hazardous agent, but the reddish skin indurations on the front chest wall were additionally confusing. Although rather unusual after gardening, the diagnosis of rhabdomyolysis was easy to establish, but the etiology remained uncertain until reliable anamnestic data were obtained. Eliciting a reliable medical history that contributes to diagnosis requires a very careful and persistent approach when psychiatric patient is involved.

## Figures and Tables

**Table 1:** The review of abnormal test results in serum (S) and urine (U).

Test	Measured value	Reference values
Creatine phosphokinase (S)	22626 U/L	<177 U/L
Aspartate transaminase (S)	443 U/L	11–38 U/L
Alanine aminotransferase (S)	194 U/L	12–48 U/L
Lactic acid dehydrogenase (S)	930 U/L	<241 U/L
Creatinine (S)	4.5 mg/dL 395 *µ*mol/L	0.9–1.4 mg/dL 79–125 *µ*mol/L
Urine myoglobin (U)	6780 *µ*g/L	<30 *µ*g/L
